# Evaluation of Pulmonary Hypoplasia in Various Congenital Anomalies with a Comparison of Two Conventional Methods of Assessment: Radial Alveolar Count (RAC) and Lung Weight: Body Weight Ratio (LBW)

**DOI:** 10.5146/tjpath.2021.01521

**Published:** 2021-05-15

**Authors:** Deepu Mathew Cherian, C. N. Sai Shalini, Chitra Andrews, Uma Maheswari, Prathiba D

**Affiliations:** Department of Pathology, Indian Institute of Medical Science and Research, Warudi, Maharashtra, India; Department of Pathology, Sri Ramachandra Institute of Higher Education and Research, Tamil Nadu, India; Department of Obstetrics and Gynecology, Sri Ramachandra Institute of Higher Education and Research, Tamil Nadu, India; Department of Neonatology and SCOPE, Sri Ramachandra Institute of Higher Education and Research, Tamil Nadu, India

**Keywords:** Congenital anomalies, Pulmonary hypoplasia, Lung weight, Body weight ratio, Radial alveolar count

## Abstract

***Objective:*** Pulmonary hypoplasia is common in the perinatal period and causes death in newborn infants. It is commonly associated with a number of malformation syndromes. Various parameters are used to estimate pulmonary hypoplasia at fetal autopsy including Lung Weight Body Weight ratio (LW:BW), Radial Alveolar Count (RAC) and DNA estimation.

***Material and Method:*** This study was carried out as a retrospective analysis of 108 lung specimens of fetuses with congenital anomalies for a period of five years. All terminated fetuses with anomalies were received with 10% formalin. An inverted Y-shaped incision was made on the fetus to remove the lungs. Lung weight and body weight were measured and the ratio was calculated. Morphometric estimation of RAC was done microscopically by counting the number of alveoli using the Q capture software. RAC was calculated based on gestational age.

***Results:*** Among the restrictive lung diseases, pulmonary hypoplasia by the LW:BW ratio was prevalent in 43% while the same by RAC was 19%. Similarly, pulmonary hypoplasia by the LW:BW ratio was prevalent in 35% while the same by RAC was 26% among cases with non restrictive lung diseases. Oligohydramnios showed the highest prevalence of pulmonary hypoplasia (23.7%), followed by renal anomalies (16.9%) and CNS anomalies (15.2%).

***Conclusion:*** Pulmonary hypoplasia is a common occurrence in many congenital anomalies, premature rupture of membranes, and hydrops fetalis. Identifying the anomaly during the intrauterine period will help to anticipate and accordingly manage the baby in the postpartum period. Early diagnosis of correctable condition like oligohydramnios will also help in the early intervention and prevention of pulmonary hypoplasia.

## INTRODUCTION

Pulmonary hypoplasia can be defined as arrested or incomplete development of the lungs. A number of growth factors are necessary for appropriate lung development, including appropriate space, normal respiratory motion, and adequate fluid, which normally distends the developing lung. Pulmonary hypoplasia can result when any one or a combination of these factors is absent or impaired (1). Pulmonary hypoplasia is common in the perinatal period and is a significant cause of death in newborn infants. Pulmonary hypoplasia in newborns is commonly associated with a number of malformation syndromes that commonly include diaphragmatic hernia and renal agenesis or dysgenesis. Other associations include skeletal muscle disorders, exomphalos, skeletal dysplasia, hydrops fetalis, trisomy 18, and prolonged rupture of the membranes.

For this study, the underlying abnormalities that may result in pulmonary hypoplasia can be categorized as either restrictive or non-restrictive types. Restrictive types include space-occupying lesions in the thorax, such as misplaced abdominal organs in congenital diaphragmatic hernia (CDH), pleural effusion, skeletal deformity, or cardiac anomaly restricting the intra-thoracic space. Non-restrictive types include oligohydramnios, urinary outflow obstruction, prolonged premature rupture of the membranes, or chromosomal anomalies (Trisomy 21). Congenital acinar dysplasia is an extremely rare primary maldevelopment of the lungs that results in pulmonary hypoplasia wherein there are multiple cystic outpouchings in each lobule lined by bronchial type epithelium, without any alveoli (2). The pulmonary hypoplasia associated with trisomy 21 is due to reduced numbers of alveoli and a smaller alveolar surface area. Therefore, hypoplastic lungs have a decreased number of airway generations, with fewer and smaller peripheral airspaces than normal.

Lungs which are hypoplastic as a result of oligohydramnios are also structurally and biochemically immature for gestational age (3). In contrast, lungs that are hypoplastic from all other causes usually have a structure that is appropriate for gestational age. The maturation arrest, which occurs with pulmonary hypoplasia due to oligohydramnios, may be specifically related to failure to retain fetal lung liquid. However, studies have shown no difference in the structure and maturity of hypoplastic lungs secondary to renal agenesis or dysplasia, compared with those associated with other types of malformations like chromosomal abnormalities and intrauterine growth retardation (4,5).

Various parameters are used to estimate pulmonary hypoplasia at fetal autopsy including the Lung Weight:Body Weight ratio (LW:BW), radial alveolar count, and DNA estimation (1,3,4). Identification of pulmonary hypoplasia is important for postnatal management. As pulmonary hypoplasia can produce a spectrum of respiratory complications, anticipating pulmonary hypoplasia will help in deciding the mode of treatment.

This study was carried out to estimate lung weight with reference to body weight (LW:BW ratio) and radial alveolar count (RAC) in the lungs of fetuses having congenital anomalies, and to compare the data in the restrictive and non-restrictive categories.

## MATERIAL and METHODS

### Study Setting and Subjects

This study was carried out as a retrospective analysis of the lung specimens of fetuses with congenital anomalies received in the Department of Pathology of our tertiary care hospital for a period of five years between 2012 and 2016. A total of 742 fetal specimens of which 227 cases had associated congenital anomalies were received for autopsy during the study period. Among these autopsies, medical records containing complete clinical information were present in 108 anomaly cases and these cases were selected for the study. Approval was obtained from the Institutional Ethics Committee prior to commencing the study. All procedures performed in the current study were approved by the IRB and/or national research ethics committee (IEC NO: CSP-MED/15/OCT/ 25/59) in accordance with the 1964 Helsinki declaration and its later amendments.

### Data Collection Tools

All terminated fetuses with anomalies were received in a plastic container with 10% formalin (9 times the volume of fetus). The umbilical cord was cut. An inverted Y-shaped incision was made on the fetus to open the thoracic and abdominal cavity. The heart and the lungs were taken out en-bloc. The lungs were separated by cutting at the hilum and were weighed separately.

#### The LW: BW Ratio

The fetus was tap dried and weighed using a CAS computing scale baby weighing scale with a minimum count of 100.000 g to record the body weight. The lungs were weighed separately using a Shimadzu BL-220H high precision electronic balance with a minimum count of 0.001 g, and the CAS SW-LR weighing scale with a minimum count of 1.000 g. The ratio of Lung weight to Body weight (LW:BW) was calculated. A LW:BW ratio < 0.018 is considered as hypoplasia (6).

#### RAC Count

A block of tissue was taken from each lobe at right angles to the direction of the main bronchus and at a distance of approximately one-third from the root of the lung to the periphery. The lung tissues were processed in the Leica ASP 300 automatic tissue processor. After tissue processing, the tissue was mounted in paraffin wax. Sections measuring 4-5 microns were made using a microtome and these sections were stained with Hematoxylin and Eosin. Morphometric estimation of radial alveolar count was done by the method proposed by Emery and Mithal (7). Under the microscope, a perpendicular line from the center of terminal bronchiole was dropped on to the nearest and definite connective tissue septum using the Q Capture software. The number of alveoli cut by this line was then counted. Ten such counts were done from each case and the average was taken as the Radial Alveolar Count (RAC). For calculating RAC, the cases were divided into 4 categories based on gestational age (Table 1) (1).

**Table 1:** Calculation of RAC based on gestational age.

**S. No**	**Gestational age**	**RAC count**	**Interpretation**
**1**	Up to 24 weeks	≥ 2	Normal
**2**	24 weeks +1 day to 30 weeks	≥ 3	
**3**	30 weeks +1 day to 25 weeks	≥ 4	
**4**	>35 weeks	≥ 5	

**RAC:** Radial alveolar count

### Data Analysis

Data were entered and analyzed using a Microsoft Excel spreadsheet. The prevalence of pulmonary hypoplasia was calculated in percentages.

## RESULTS

This study was carried out among 108 specimens received for autopsy at the Department of Pathology. Out of the 108 specimens, 42 fetuses were of the restrictive type and 66 fetuses of the non-restrictive type. Of the restrictive type, 26 cases had a cardiac anomaly, nine cases had a skeletal anomaly, and seven cases had pulmonary anomalies. Of the non-restrictive type, seven cases had abdominal wall defects, six cases had chromosomal anomalies, 16 cases had central nervous system anomalies, 21 cases were of anhydramnios/oligohydramnios, 10 cases were of renal anomalies, 4 cases had hydrops fetalis, 1 case had the amniotic band syndrome, and 1 cases had a storage disorder (beta glucosidase deficiency) (Table 2).

**Table 2:** Background characteristics of the cases.

**S. No**	**Characteristics**	**Frequency (n=108)**	**Percentage (%)**
1	Type of Congenital Anomaly		
	Restrictive	42	38.9
	Non restrictive	66	61.1
2	Organ involvement among restrictive category (n=42)		
	Cardiac anomaly	26	61.9
	Skeletal anomaly	9	21.4
	Pulmonary anomaly	7	16.7
3	Organ involvement among non restrictive category (n= 66)		
	Abdominal wall defect	7	10.6
	Chromosomal anomalies	6	9.1
	Central nervous system defect	16	24.2
	Oligo/anhydramnios	21	31.8
	Renal defects	10	15.2
	Hydrops fetalis	4	6.1
	Amniotic band syndrome	1	1.5
	Storage diseases	1	1.5

The prevalence of pulmonary hypoplasia is given in Table 3. Among the restrictive lung diseases, pulmonary hypoplasia by the LW:BW ratio was prevalent in 43% while the same by RAC was 19%. Similarly, pulmonary hypoplasia by the LW: BW ratio was prevalent in 35% while the same by RAC was 26% among cases with non-restrictive lung diseases.

**Table 3:** Prevalence of pulmonary hypoplasia among the cases.

**S. No**	**Categories**	**Frequency**	**Percentage (%)**
**1**	**Restrictive category (n=42)**		
	***LW:BW ratio <0.018***	***18***	***42.85***
	Cardiac Anomalies	8	19
	Pulmonary anomalies	5	11.9
	Skeletal Anomalies	5	11.9
	***RAC abnormal***	***8***	***19***
	Cardiac Anomalies	6	14.3
	Pulmonary Anomalies	1	2.4
	Skeletal Anomalies	1	2.4
**2**	**Non restrictive category(n=66)**		
	***LW:BW<0.018***	***43***	***65.1***
	Storage disorders	1	1.5
	Miscellaneous	5	7.6
	Renal Anomalies	10	15.1
	PPROM/Oligohydramnios	14	21.2
	CNS Anomalies	9	13.6
	Chromosomal Anomalies	3	4.5
	Abdominal wall defects	1	1.5
	***RAC abnormal***	***17***	***25.7***
	Renal Anomalies	1	1.5
	Abdominal Wall Defects	1	1.5
	PRROM/Oligohydramnios	6	9
	CNS Anomalies	3	4.5
	Chromosomal Anomalies	3	4.5
	Miscellaneous	3	4.5

**LW:BW:** Lung weight: Body weight ratio, **RAC:** Radial alveolar count, **PPROM:** Preterm premature rupture of the membranes, **CNS:** Central nervous system

The system-wide prevalence of pulmonary hypoplasia based on the :BW ratio is given in Table 4. Oligohydramnios showed the highest prevalence of pulmonary hypoplasia (23.7%), followed by renal anomalies (16.9%) and CNS anomalies (15.2%).

**Table 4:** System-wise prevalence of pulmonary hypoplasia based on the LW:BW ratio.

**S. No**	**Characteristic**	**Hypoplasia**		**Normal**		**Total (n=108)**
		**N (61)**	**%**	**N (47)**	**%**	**N(%)**
1	Storage disorders	1	(1.6)	0	(0.0)	1 (0.9)
2	Miscellaneous	5	(8.2)	0	(0.0)	5 (4.6)
3	Renal Anomalies	10	(16.4)	0	(0.0)	10 (9.3)
4	Pulmonary anomalies	5	(8.2)	2	(4.3)	7 (6.5)
5	PPROM/Oligohydramnios	14	(23.0)	7	(14.9)	21 (19.4)
6	CNS Anomalies	9	(14.8)	7	(14.9)	16 (14.8)
7	Skeletal Anomalies	5	(8.2)	4	(8.5)	9 (8.3)
8	Chromosomal Anomalies	3	(5.0)	3	(6.4)	6 (5.6)
9	Cardiac Anomalies	8	(13.1)	18	(38.3)	26 (24.1)
10	Abdominal wall defects	1	(1.6)	6	(12.8)	7 (6.5)

**PPROM:** Preterm premature rupture of the membranes, **CNS:** Central nervous system

The system-wide prevalence of pulmonary hypoplasia based on RAC is given in Table 5. The highest prevalence was observed with oligohydramnios and cardiac anomalies (24%), followed by other anomalies like CNS and chromosomal anomalies (12%).

**Table 5:** System-wise prevalence of pulmonary hypoplasia based on RAC.

**S. No**	**Characteristic**	**RAC <Normal**		**RAC Normal**			**N(108)**
		**N (25)**	**%**	**N (83)**		**%**	
1	Storage disorder	0	(0.0)	1	(1.2)		1 (1.0)
2	Renal Anomalies	1	(4.0)	9	(10.8)		10 (9.3)
3	Skeletal Anomalies	1	(4.0)	8	(9.6)		9 (8.3)
4	Abdominal Wall Defects	1	(4.0)	6	(7.2)		7 (6.5)
5	Cardiac Anomalies	6	(24.0)	20	(24.1)		26 (24)
6	PRROM/ Oligohydramnios	6	(24.0)	15	(18)		21 (19.4)
7	CNS Anomalies	3	(12.0)	13	(15.7)		16 (14.8)
8	Pulmonary Anomalies	1	(4.0)	6	(7.2)		7 (6.5)
9	Chromosomal Anomalies	3	(12.0)	3	(3.6)		6 (5.6)
10	Miscellaneous	3	(12.0)	2	(2.4)		5 (4.6)

**PPROM:** Preterm premature rupture of the membranes, **CNS:** Central nervous system

## DISCUSSION

Pulmonary hypoplasia, as defined by Boyden, is a maldevelopment of lungs in which normal pulmonary tissue is present but is underdeveloped. Pulmonary hypoplasia is noted in more than 10% of neonatal autopsies and occurs in association with another malformation (or malformations) in more than 85% of the cases (8). The most frequently occurring anomalies are diaphragmatic defects and renal malformations, but a wide variety of anomalies have been described. The common feature of most of these anomalies is that they directly compromise the thoracic space available for lung growth. *In utero *accumulation of fluid within the thorax as pleural effusion or chylothorax has also been implicated in the production of pulmonary hypoplasia. Pulmonary hypoplasia may also occur in the absence of other anomalies or in cases of preterm premature rupture of amniotic membranes. As with infants with hypoplasia secondary to other anomalies, these infants present with respiratory distress, are difficult to ventilate, and frequently have episodes of pneumothorax and interstitial pulmonary emphysema.

The abdominal wall is an integral component that determines the thoracic cavity dimensions. Defects in the ventral abdominal wall alter respiratory mechanics and can impair diaphragm function. Congenital abdominal wall defects also are associated with abnormalities in lung growth and development that lead to pulmonary hypoplasia, pulmonary hypertension, and alterations in thoracic cage formation (9). The findings of our study showed the evidence of arrest in alveolar development in cases of abdominal wall defect with normal lung parenchymal developmental maturation. This was in concordance with the study conducted by J. Craig Argyle (10).

The mean LW:BW value was 0.036 ± 0.01 and the RAC count was 2.54±0.5. This was in concordance with the study by Askenazi and Perlman (6). Reale and Esterly (11) showed a mean LW:BW ratio of 0.013 and RAC of 4.6. All the cases of renal anomalies showed a LW:BW ratio <0.018. This was in concordance with the study conducted by Monique (12), Askenazi and Perlman (6), and Husain and Hessel (1).

Pulmonary hypoplasia was evidenced in 66.7% of the cases with anhydramnios and premature rupture of membranes, similar to studies done by Wigglesworth (13). Out of the four cases of hydrops fetalis, pulmonary hypoplasia was present in 100%, similar to studies by Askenazi (13) and Hussain (1). This establishes the strong association between lung hypoplasia and hydrops fetalis. None of the 4 cases were associated with pleural effusion which suggests that the reduction in the thoracic cavity is not the primary cause of lung hypoplasia.

Pleural effusion in the intrauterine period is a predictor of pulmonary hypoplasia. If the fluid collection occurs before the 24th to the 26th of gestation, it produces irreversible damage whereas when it occurs in the alveolar stage, it may not produce irreversible damage (14,15). Pleural effusions occurring in the mid-trimester can be detected by antenatal scans and definitive *in utero* therapy can be considered when no other major fetal abnormality is detected. Decompression of the thorax either by repeated transfusions or placing a pleural amniotic shunt can be tried in such cases in the fetus (16,17). Our study demonstrated 100% pulmonary hypoplasia among fetuses with pleural effusion, similar to the study conducted by Castillo (18).

Development of the lung is intrinsically related to that of the heart. An association between pulmonary hypoplasia and congenital heart diseases has previously been suggested based on postnatal studies. Autopsy studies have shown that the affected infants have an abnormally small number of alveoli (19,20). The impaired alveolarization in patients with right outflow obstruction may be an important cause of the low lung volumes measured in children and adults operated for the tetralogy of Fallot (21–25). About 13% of congenital heart disease cases showed pulmonary hypoplasia in a study conducted by Isabelle (26) but this value was 30.8% in our study.

The LW:BW ratio detected a greater number of anomaly cases compared to RAC. Identification of pulmonary hypoplasia by both the methods was almost similar in case of cardiac anomalies. LW:BW showed 8 cases with pulmonary hypoplasia while RAC showed 6 cases with pulmonary hypoplasia. The LW:BW ratio detected 5 cases of pulmonary hypoplasia in lung anomalies and skeletal anomalies, while RAC detected only 1 case of pulmonary hypoplasia in lung anomalies and skeletal anomalies.

It is well established that renal anomalies produce pulmonary hypoplasia. The LW:BW ratio could detect 10 cases of pulmonary hypoplasia in renal anomalies. On the other hand, RAC detected only one case of pulmonary hypoplasia among renal anomaly cases. Detection of pulmonary hypoplasia cases was more common by LW:BW in preterm premature rupture of the membranes (PPROM)/Oligohydramnios and CNS anomalies, with 14 and 9 cases respectively detected, while RAC detected 6 and 3 cases, respectively.

In conclusion, our study demonstrated that PPROM/Anhydramnios was the most common cause of pulmonary hypoplasia. Renal anomalies and Hydrops fetalis are strongly associated with pulmonary hypoplasia. Abdominal wall defect and cardiac anomalies are less frequent causes of pulmonary hypoplasia. We conclude that pulmonary hypoplasia is a common occurrence in many congenital anomalies, premature rupture of membranes, and hydrops fetalis. Identifying the anomaly during the intrauterine period will help to anticipate and accordingly manage the baby in the postpartum period. Early diagnosis of correctable defects like oligohydramnios will also helps in the early intervention and prevention of pulmonary hypoplasia. This study also highlights the significance of performing fetal autopsy, especially in case of congenital anomalies.

## Conflict of INTEREST

The authors declare that they have no potential conflicts of interest to disclose.

## FUNDING

None.
